# Psychometric properties of the original and short versions of the Falls Efficacy Scale-International (FES-I) in people with Parkinson’s disease

**DOI:** 10.1186/s12955-017-0689-6

**Published:** 2017-05-31

**Authors:** Stina B Jonasson, Maria H Nilsson, Jan Lexell

**Affiliations:** 1 Department of Health Sciences, Lund University, Box 157, 221 00 Lund, Sweden; 2 Department of Neurology and Rehabilitation, Skåne University Hospital, Lund, Sweden; 3 Memory Clinic, Skåne University Hospital, Malmö, Sweden; 4 Department of Health Science, Luleå University of Technology, Luleå, Sweden

**Keywords:** Concerns about falling, Parkinson disease, Psychometrics, Reliability of results

## Abstract

**Background:**

Fear of falling is common in people with Parkinson’s disease (PD) and is associated with an increased risk for future falls, activity limitations and a reduced quality of life. The Falls Efficacy Scale-International (FES-I) assesses fear of falling conceptualized as concerns about falling. The original FES-I has good psychometric properties in people with PD, but whether this applies also for the short version of FES-I remains to be shown.

The aim of the present study was to evaluate the psychometric properties of the short FES-I and to compare these with the original FES-I in the same sample of people with PD. The investigated psychometric properties included known groups validity, data completeness, scaling assumptions, targeting and reliability.

**Methods:**

A postal survey, which included the original, full-length FES-I, was distributed to 174 people with PD. Responders received a second survey after two weeks. From these data, short FES-I total scores were calculated by extracting the items that are included in the short version of the scale.

**Results:**

Median age and PD duration of the 101 responders (43% women) were 73 and 5 years, respectively. The original as well as the short FES-I scores were able to discriminate (*p* < 0.001) between groups with and without fear of falling, activity avoidance, falls, near falls, and with various self-rated PD severity, respectively. Both versions of FES-I had a high level of data completeness (0.7 to 0.9% missing item responses). Scaling assumptions were acceptable for the original as well as the short FES-I. While the short FES-I had 19% floor effect, the original version was better targeted. Both versions were reliable and obtained high values for internal consistency (Cronbach’s alpha >0.8) and test-retest reliability (Intraclass Correlation Coefficient > 0.9).

**Conclusions:**

Both the original and short FES-I revealed generally good psychometric properties in people with PD, although the original scale was better targeted. Due to the higher floor effect in the short FES-I, the present findings favors using the original, full-length FES-I in longitudinal follow-ups, intervention studies and clinical practice when addressing concerns about falling.

## Background

Fear of falling (FOF) is common in people with Parkinson’s disease (PD) and is associated with an increased risk for future falls, activity limitations and a reduced quality of life [1–3]. FOF has been defined as “a lasting concern about falling that leads to an individual avoiding activities that he/she remains capable of performing” [4]. As of today, there is no established method for preventing or reducing FOF in people with PD. Appropriate and high quality rating scales that target FOF are essential when developing and evaluating such interventions.

Recently, we published a head-to-head comparison of the psychometric properties of four commonly used FOF rating scales in people with PD [5]. The study revealed good psychometric properties (data completeness, scaling assumptions, targeting and reliability) of the Falls Efficacy Scale-International (FES-I), which assesses FOF conceptualized as concerns about falling [6]. FES-I revealed high data completeness and test-retest reliability, which indicate that the scale includes items that are relevant and understood by people with PD and that it can be used for groups as well as for individuals with PD.

A short version of FES-I has been developed to satisfy the desire for a shorter instrument [7]. The short version requires less time to respond compared to the original version, which is advantageous in research as well as in clinical context. The original version has obtained high internal consistency (Cronbach’s alpha 0.96) in older people [6] as well as in people with PD [5], which might be a sign of item redundancy [8]. The short FES-I has shown good psychometric properties in older people [7]. However, to the best of our knowledge, no study has assessed its psychometric properties in people with PD. A better understanding of the psychometric properties of the short version of FES-I will allow us to determine its suitability in clinical practice and research.

The aim of the present study was to evaluate the psychometric properties of the short FES-I in people with PD and to compare these with the original version of FES-I. We assessed and compared data completeness, scaling assumptions, targeting and reliability for both versions of the scale. Moreover, this study expands our understanding of the measuring properties of FES-I by including analyses of known groups validity of the two scales, i.e., if they were able to discriminate between groups that were hypothesized to report high and low concerns about falling, respectively.

## Methods

### Research design

The current study is a secondary analysis of a cross-sectional survey conducted with 174 participants with PD [5]. The previously published study included psychometric analyses of the original, full-length FES-I. From that dataset, we extracted data for the items that are included in the short FES-I and could then calculate total scores for the original as well as the short FES-I, which enabled a head-to-head comparison.

### Participants and recruitment

**Fig. 1:**
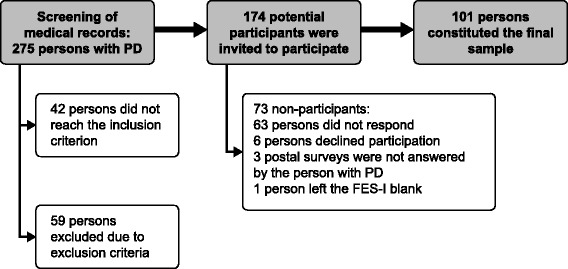
Flow chart of the recruitment process. *PD* Parkinson’s disease; *FES-I* Falls Efficacy Scale-International

Participants were recruited from two out-patient clinics in southern Sweden. Inclusion criterion was a PD diagnosis (ICD-10: G 20.9) since at least one year. Exclusion criteria were difficulties reading and writing Swedish, clinically confirmed dementia, or cognitive or medical problems of a severity that were assumed to restrict giving informed consent or participating in the study. Moreover, those who were completely bedridden or wheelchair bound were excluded. A PD specialized nurse at each outpatient clinic and one of the authors (SBJ) screened the medical records for all PD patients that had visited the two clinics during the past 14 months (*n* = 275). After applying the inclusion and exclusion criteria, 174 potential participants remained. A flow chart of the recruitment process is presented in Fig. 1.

### Procedure

The 174 potential participants received a postal survey, which included information about the study, a written informed consent form, demographic and disease-related questions, FES-I and a pre-stamped return envelope. FES-I was administered at two test occasions (test and retest, hereafter referred to as t1 and t2), two weeks apart. A reminder was sent to non-responders after two weeks at t1, and after one week at t2.

### Assessments

#### Demographic and disease-related questions

The postal survey at t1 included questions about PD duration, self-rated PD severity (mild/moderate/severe) and living arrangements (alone/not alone). Dichotomous questions (yes/no) targeted FOF (“Are you afraid of falling?”), activity avoidance due to the risk of falling (“Do you avoid activities due to the risk of falling?”), use of mobility devices indoors and outdoors, respectively, and falls and near falls during the past six months, respectively. A fall was defined as “an event in which the respondent came to rest on the ground, floor, or lower level” (definition adopted from the Prevention of Falls Network Europe) [9]. A near fall was defined as “a fall initiated but arrested by support from a wall, railing, or other person, etc.” [10]. The Parkinson’s disease Activities of Daily Living Scale was used to assess difficulties in activities of daily living (possible response categories: no/mild/moderate/high levels of/extreme difficulties with day-to-day activities [11]). Finally, participants were asked whether they had responded to the survey themselves (with or without assistance in reading and/or writing).

The postal survey at t2 included questions if the participants’ PD treatment had changed since t1 and whether they considered that their level of FOF, fall frequency, balance or walking abilities had changed since t1.

#### Concerns about falling

The Swedish translated, full-length FES-I [12] was included to assess concerns about falling [6]. Respondents answer the overall question how concerned they are about the possibility of falling in relation to different activities (items), e.g., taking a bath or shower, and going out to a social event. The original FES-I contains 16 different items, whereof seven of these are included in short FES-I (i.e., item number 2, 4, 6, 7, 9, 15 and 16) [7]. The response options are: Not at all concerned, Somewhat concerned, Fairly concerned, or Very concerned (scored 1 to 4, respectively). For both versions, a total score (higher = worse) is calculated by summing the included items. The total score of the original FES-I ranges from 16 to 64 [6] and can be categorized into groups describing low (16–19 points), moderate (20–27 points) and high concerns about falling (28–64 points) [13]. The total score for the short FES-I ranges from 7 to 28 [7, 12].

### Data analysis

All statistical analyses were performed in IBM SPSS Statistics, version 24, and were based on five parts: i) known groups validity, ii) data completeness, iii) scaling assumptions, iv) targeting, and v) reliability. The relationship between the original and short FES-I was determined by calculating the Spearman’s correlation coefficient (*r*_*s*_). Data completeness and reliability (except Cronbach’s alpha) were based on data from both t1 and t2, whereas the other analyses were based on t1 data only. Two-tailed *p*-values were used, and the level of statistical significance was set to *p* < 0.05.

#### Known groups validity

Known groups validity was studied by investigating whether the rating scales could distinguish between groups that are hypothesized to differ in levels of concerns about falling [14]. Non-parametric group comparisons (Mann-Whitney U-test and Kruskal-Wallis test, respectively) were used to investigate if the original and short FES-I were able to separate people with and without FOF, activity avoidance due to the risk of falling, falls and near falls during the past six months, respectively, and people with different self-rated PD severity.

Previous studies have shown that concerns about falling is strongly correlated with fall-related self-efficacy and fall-related activity avoidance in people with PD [5]. Thus, we hypothesized that those who affirmed the dichotomous questions on FOF and activity avoidance due to the risk of falling would obtain higher FES-I total score. Kader et al. showed that fall-related activity avoidance is significantly more extensive in people with severe PD than among those with less severe PD, and among those who have a history of falls or near falls than among those without such history [2]. As concerns about falling and fall-related activity avoidance are closely related [5], we hypothesized that the same patterns would apply also for concerns about falling. That is, those who rated their PD as more severe and those who affirmed the dichotomous questions targeting previous falls and near falls, respectively, were hypothesized to be more concerned about falling.

#### Data completeness

Data completeness is a measure of the degree to which a rating scale is completed. The percentage of missing data was investigated on item as well as total score level [15, 16]. Data completeness has been suggested to be adequate if missing data is less than 10% [17]. Imputation was not used, i.e., only participants who responded to all items in a rating scale obtained a total score on that scale.

#### Scaling assumptions

Scaling assumptions refer to the legitimacy of summing items into a total score. This was evaluated by studying a set of criteria. Mean scores, standard deviations and distribution of item response option frequency should be roughly parallel across items. Moreover, corrected item-total correlations should exceed 0.4, which indicate that items measure the same underlying construct and contribute with enough information to the total score [15, 16].

#### Targeting

A well-targeted rating scale is characterized by a score distribution that is able to represent the true level of concerns about falling in the study sample [15]. This implies that mean total scores should be close to the scales’ midpoints [18] (i.e., original FES-I 40; short FES-I 17.5), total scores should range the full span of possible scale scores [18] (i.e., original FES-I 16–64; short FES-I 7–28), skewness should be less than ±1 [18] and floor and ceiling effects should be smaller than 15–20% [15, 18, 19].

#### Reliability

Reliability refers to the random error that is associated with scale scores and whether the score can be reproduced if the level of concerns about falling is unchanged [15]. Multiple measures were used to assess the reliability. Cronbach’s alpha was used as a measure of the internal consistency of the rating scales [20]. One-way random, single measures Intraclass Correlation Coefficient (ICC) was used as a measure of the test-retest reliability [21]. Cronbach’s alpha and ICC values above 0.75 or 0.80 are suggested to be acceptable for using rating scales on group level [22, 23], whereas ICC >0.90 has been suggested as the minimum when a rating scale is used for individual comparisons [23, 24]. The standard error of measurement (SEM) was calculated using the formula *SD*_*baseline*_ $\times \sqrt{1- reliability}$ [25]. Since there is no consensus whether Cronbach’s alpha or ICC should be used as the reliability coefficient in the SEM formula [24, 25], both versions were calculated. The smallest detectable difference (SDD) was calculated using the formula $SEM\times 1.96\times \sqrt{2}$ [26]. To enable comparisons between the original and short FES-I, which have different scoring ranges, SEM and SDD were also expressed as percentages of the scales’ possible scoring ranges. Finally, the number of outliers in each rating scale was calculated, i.e., individuals with large differences in scale scores between t1 and t2. An outlier was defined as a participant with a difference in scale scores between t1 and t2 outside the first or third quartile ±1.5 × interquartile range of the mean difference in scale scores between t1 and t2 [27].

## Results

Out of the 174 potential participants, 63 persons did not respond the postal survey, 6 persons declined participation, 3 postal surveys were not answered by the person with PD and 1 person had left the FES-I blank. This resulted in a final study sample of 101 persons (43% women). Their median (first-third quartile; min-max) age was 73 (68–78; 52–91) years and PD-duration was 5 (3–11; 1–30) years. See Table 1 for further participant characteristics.

**Table 1:** Participant characteristics

Gender (women)	43/101
Age (years), median (first-third quartile)	73 (68–78)^a^
Parkinson duration (years), median (first-third quartile)	5 (3–11)^b^
Self-rated Parkinson severity	
Mild	24/97
Moderate	61/97
Severe	12/97
Fear of falling (yes)	55/101
Activity avoidance due to risk of falling (yes)	53/101
Concerns about falling^1^	
Low (16–19 points)	23/92
Moderate (20–27 points)	27/92
High (28–64 points)	42/92
Falls past 6 months (yes)	35/101
Near falls past 6 months (yes)	55/101
Use of mobility devices indoors/outdoors, respectively (yes)	24/95 and 42/96
Need help from others in daily activities (yes)^2^	18/91
Living alone (yes)	27/100

Data are n/total unless otherwise stated

^1^Falls Efficacy Scale-International

^2^Parkinson’s disease Activities of Daily Living Scale. Dichotomized: “No” and “Mild difficulties with day-to-day activities” are recorded as “No”. “Moderate”, “High levels of” and “Extreme difficulties with day-to-day activities” are recorded as “Yes”

^a^ *n* = 101

^b^ *n* = 97

In comparison to the 73 non-participants (median age 79 years, 55% women), those in the final study sample were significantly younger (*p* = 0.002) but there was no statistical difference in gender (*p* = 0.111).

The relationship between the total score of the original and short-FES-I was *r*_*s*_ = 0.97, *p* < 0.001.

### Known groups validity

The discriminant capacity of the original and short FES-I is presented in Table 2. Both versions of the scale were able to discriminate between the various groups as hypothesized. That is, those who were afraid of falling, avoided activities, had experienced falls and near falls during the past six months, respectively, and those who rated their PD as more severe were more concerned about falling, and obtained significantly (*p* < 0.001) higher total scores on the original as well as the short FES-I.

**Table 2:** Discriminant capacity of the original and short FES-I

	**Fear of falling**			
	No	Yes		*p*
Original FES-I	19 (17–24)	36 (26–47)		<0.001
Short FES-I	8 (7–10)	26 (12–20)		<0.001
	**Activity avoidance**			
	No	Yes		*p*
Original FES-I	20 (17–25)	37 (26–47)		<0.001
Short FES-I	8 (7–11)	16 (12–20)		<0.001
	**Falls past six months**			
	No	Yes		*p*
Original FES-I	22 (18–30)	38 (27–48)		<0.001
Short FES-I	9 (7–13)	17 (12–20)		<0.001
	**Near falls past six months**			
	No	Yes		*p*
Original FES-I	22 (17–25)	32 (24–46)		<0.001
Short FES-I	9 (7–12)	14 (10–20)		<0.001
	**Self-rated Parkinson severity**			
	Mild	Moderate	Severe	*p*
Original FES-I	20 (17–23)	29 (20–41)	45 (30–54)	<0.001^*^
Short FES-I	8 (7–9)	12 (9–17)	20 (13–23)	<0.001^*^

Data are presented as median (first-third quartile)

*P*-values are based on Mann-Whitney U-tests, except the *p*-values for Self-rated Parkinson severity, which are based on Kruskal-Wallis test

*FES-I* Falls Efficacy Scale-International. Possible scoring ranges: Original 16–64, Short 7–28; higher = worse

*All subsequent unpaired comparisons showed statistical significant differences (Bonferroni correction criterion of *p* < 0.016), except between persons with Moderate and Severe Parkinson severity for Short FES-I (*p* = 0.017)

### Data completeness

At t1, 92 of the 101 participants had responded to all original FES-I items and obtained a total score. The corresponding number for the short FES-I was 96 participants. The mean of missing item responses was 0.9% for the original FES-I whereas it was 0.7% for the short FES-I. At t2, 89 and 92 participants obtained a total score for the original and short FES-I, respectively. Missing responses on item level are presented in Table 3.

**Table 3:** Scoring distribution and data completeness of Falls Efficacy Scale-International (FES-I)

Item	Activity	Mean (SD) *n* = 101	Missing or invalid responses
1	Cleaning the house	2.0 (1.1)	2
**2**	**Getting dressed or undressed**	1.5 (0.8)	1
3	Preparing simple meals	1.4 (0.8)	2
**4**	**Taking a bath or shower**	1.6 (1.0)	-
5	Going to the shop	1.7 (1.1)	1
**6**	**Getting in or out of a chair**	1.7 (0.8)	2
**7**	**Going up or down stairs**	2.0 (1.0)	1
8	Walking around in the neighbourhood	1.7 (0.9)	-
**9**	**Reaching for something above your head or on the ground**	2.0 (1.0)	1
10	Going to answer the telephone before it stops ringing	1.7 (0.9)	2
11	Walking on a slippery surface	2.7 (1.0)	-
12	Visiting a friend or relative	1.7 (0.9)	1
13	Walking in a place with crowds	1.9 (1.0)	-
14	Walking on an uneven surface	2.3 (1.1)	1
**15**	**Walking up or down a slope**	2.3 (1.1)	-
**16**	**Going out to a social event**	1.7 (0.9)	-

Possible item score range 1–4, higher = worse

Bold items are included in Short FES-I

### Scaling assumptions

Item means, SDs and response option frequency were roughly parallel for most items. However, item 3 (Preparing simple meals) had a higher proportion of participants that chose the best response option, resulting in a lower item mean score (i.e., easier item). Items 11 (Walking on a slippery surface), 14 (Walking on an uneven surface) and 15 (Walking up or down a slope) had a larger proportion of participants that chose the worse response categories, resulting in higher item mean scores (i.e., more difficult items). Out of these, only item 15 is included in short FES-I. All corrected item-total correlations exceeded 0.4 for both the original and short FES-I (Table 4).

**Table 4:** Psychometric comparison of the original and short FES-I, *n* = 101

	Original FES-I	Short FES-I
Missing item responses	0.9%	0.7%
Corrected item-total correlation, min-max	0.59 (item 6)–0.85 (items 1 and 7)	0.63 (item 16)–0.78 (item 7)
Total scores, *n*	92	96
Mean (SD)	30 (12.0)	13 (5.1)
Min-Max	16–59	7–25
Skewness	0.72	0.71
Floor/ceiling effects	10/0	19/0
Cronbach’s alpha	0.96	0.89
ICC (95% CI)	0.92 (0.88–0.95)	0.91 (0.86–0.94)
SEM^a^ (% of possible scoring range)	3.4 (7)	1.6 (7)
SEM^b^ (% of possible scoring range)	2.5 (5)	1.7 (8)
SDD^c^ (% of possible scoring range)	9.6 (20)	4.3 (20)
SDD^d^ (% of possible scoring range)	6.8 (14)	4.7 (21)

*FES-I* Falls Efficacy Scale-International, *SEM* Standard Error of Measurement, *SDD* Smallest Detectable Difference

Possible scoring ranges: Original FES-I 16–64, Short FES-I 7–28; higher = worse

^a^Based on ICC, using the formula *SEM = SD*_*baseline*_ $\times \sqrt{1- ICC}$

^b^Based on Cronbach’s alpha, using the formula *SEM = SD*_*baseline*_ $\times \sqrt{1- Cronbac{h}^{\prime } s\  alpha}$

^c^Based on the ICC-based SEM, using the formula $SDD= SEM\times 1.96\times \sqrt{2}$

^d^Based on the Cronbach’s alpha-based SEM, using the formula$SDD= SEM\times 1.96\times \sqrt{2}$

### Targeting

Total scores spanned almost the full range of possible scale scores for the original as well as the short FES-I. Mean scores were fairly close to the scales’ midpoints (within 1 SD), skewness was <±1 and floor and ceiling effects were <20% for both scales (Table 4).

### Reliability

Stability of the participants’ condition were studied by analyzing responses to the questions at t2 regarding changes in PD treatment, or perceived changes in level of FOF, fall frequency, balance or walking abilities since t1. For those participants who indicated that there had been changes, the authors manually screened the FES-I data for any significant discrepancies between t1 and t2. As no discrepancies were found that differed from the participants who did not report any changes, this did not result in any exclusion of participants.

The mean time between responses to the FES-I at t1 and t2 was 16.7 (SD 3.8, min-max 13–38) days. Cronbach’s alpha was well above 0.80 and ICC was above 0.90 for both the original and short FES-I. SEM and SDD values are presented in Table 4. There were four outliers in the original FES-I and six outliers in the short FES-I.

## Discussion

This is, to the best of our knowledge, the first study that evaluates the psychometric properties of the short FES-I in people with PD. Our study is based on data from a previously published psychometric study of the original, full-length FES-I in people with PD [5]. Thus, the psychometric properties of the original FES-I have already been reported (except for the known groups validity) and are included in this study for comparative reasons only. The results reveal generally good psychometric properties for both the original and short version of FES-I.

There was a very strong correlation (*r*_*s*_ = 0.97) between the original and short FES-I, which is not surprising as all items from the short FES-I are also included in the original FES-I. Although the discriminant capacity was as hypothesized for both the original and short FES-I, it needs to be noted that this is just one aspect of validity. Data completeness was excellent for the original as well as the short FES-I, which suggest that items were perceived as relevant and understandable by the participants [16].

Four out of the 16 items in the original FES-I were not parallel in terms of response option frequency compared to the other items in the scale. Three out of these items induced higher concerns about falling and one item induced lower concerns compared to the other FES-I items. Only one of these items is included in the short FES-I. It should be noted that the item that induced the highest concerns about falling (“Walking on a slippery surface”) in the present study sample as well as in older persons without PD [6] is not included in the short FES-I. This might partly explain the relatively high floor effect of the short FES-I in the present study. When developing the short FES-I, the authors strived to include items that provoked very low, medium and very high concerns about falling, respectively [7]. Only one out of the three most “difficult” items according to the participants in the present study is included in short FES-I, whereas two out of the three “easiest” items are included. While classical test theory suggests that items within a scale should be roughly parallel in order to use a summed total score [15, 16], no guidelines exist that describe how rigid this judgment should be. The corrected item-total correlations exceeded 0.4 for all items in the short as well as the original FES-I. This indicates that items measure the same underlying construct and contribute with enough information to the total score, which supports the use of summed total scores for both versions [15, 16].

Both the original and short FES-I were fairly well targeted, but the floor effect of the short version was almost twice as high as for the original version (19% vs 10%). While still below the recommended 20% [15], the result implies that almost one fifth of the participants obtained the best possible score on the short version of FES-I. Thus, the scale would not be capable of detecting any further reductions of concerns about falling in this subgroup of the study sample, which limits the scale’s usability in longitudinal follow-ups. Importantly, these results favor the use of the original FES-I in intervention studies and clinical practice when addressing concerns about falling.

Internal consistency and test-retest reliability was good for both versions of the scales; both scales met the suggested criteria for usage on group as well as individual level (Cronbach’s alpha >0.80 and ICC >0.90) [22–24]. Test-retest reliability was almost identical for the two versions (ICC 0.92 vs. 0.91), whereas the original FES-I obtained somewhat higher internal consistency (Cronbach’s alpha 0.96 vs. 0.89). This difference is likely due to the fact that the original version contains more items [8]. SEM% and SDD% that were based on ICC were identical for both versions of FES-I, whereas SEM% and SDD% that were based on Cronbach’s alpha were lower (i.e., better) for the original FES-I. Applying the numbers that are based on ICC values, these suggest that a change in the mean FES-I total score of more than 7% of the possible scoring range indicates a real change (i.e., above measurement error) on a group level for the original as well as short FES-I. Similarly, a changed total score for an individual of more than 20% of the possible scoring range indicates a real change (i.e., above measurement error) in concerns about falling for that individual.

There are some limitations of the present study. The postal survey study design implies that FES-I was self-administered. It needs to be underlined that the present findings may not apply if the scale is instead administered as an interview. The present study included administration of the full-length FES-I only, which can be considered a weakness as well as a strength of the study. Administration of the short FES-I might result in slightly altered responses, compared to extracting the items from the full-length version of the scale, as done in the present study. However, one could argue that using only one version of the scale enabled a fair head-to-head comparison of their psychometric properties, as analyses were based on the same data set.

There is a need for additional studies of the psychometric properties of the original as well as the short FES-I. To the best of our knowledge, the present study and our previously published study of the original FES-I (which is based on the same data set) [5] are the only psychometric studies of FES-I in people with PD. As psychometric properties are sample dependent [15], additional studies are required to confirm the psychometric properties of the rating scale in people with PD. Moreover, studies of other psychometric aspects, such as responsiveness, and studies using modern psychometric methods, such as Rasch analysis, are warranted.

## Conclusions

Both the original and short FES-I revealed generally good psychometric properties in people with PD, although the original scale was better targeted. Due to the higher floor effect in the short FES-I, the present findings favors using the original, full-length FES-I in longitudinal follow-ups, intervention studies and clinical practice when addressing concerns about falling.

## Abbreviations

FES-I: Falls Efficacy Scale-International
FOF: Fear of falling
ICC: Intraclass correlation coefficient
PD: Parkinson’s disease
SDD: Smallest detectable difference
SEM: Standard error of measurement
